# Efficacy of chitinases from mangrove wetland derived *Penicillium oxalicum* on powdered chitin

**DOI:** 10.3389/fmicb.2026.1773725

**Published:** 2026-03-18

**Authors:** Biaoshi Wang, Yalin Zhang, Jianzhi Ye, Jie Ma, Shuyan Liu, Wenjie Ou, Lulu Cao, Edward S.X. Moh, Zhiping Han

**Affiliations:** 1 College of Food Science and Engineering, Lingnan Normal University, Guangdong, China; 2 Agricultural Products Processing Research Institute, Chinese Academy of Tropical Agricultural Sciences, Guangdong, China; 3 College of Food Science and Technology, Huazhong Agricultural University, Hubei, China; 4 ARC Center of Excellence for Synthetic Biology, Macquarie University, School of Natural Sciences, Sydney, NSW, Australia

**Keywords:** chitin powder degradation, chitinase mixture, GlcNAc production, mangrove fungi, *Penicillium oxalicum*

## Abstract

Chitin, an abundant polysaccharide in shrimp and crab shells, serves as a primary resource for producing glucosamine and chitosan oligosaccharides. Chemical hydrolysis is widely utilized in these processes, due to the limited efficacy of many chitinases on powdered chitin. Additionally, many chitinases perform much better on colloidal chitin, which still requires swelling of the powdered chitin with hydrochloric acid. In this study, chitinases from a mangrove isolated *P. oxalicum* were characterized and applied for the hydrolysis of chitin substrates. The enzyme mixture exhibited strong catalytic performance toward powdered chitin, achieving approximately 70% hydrolysis within 2 h at 65°C under scale-up conditions (40 L working volume in 50 L batch reactors). HPLC analysis confirmed a stepwise hydrolysis pattern from chitooligosaccharides to monomeric GlcNAc, indicating near-complete depolymerization. The GlcNAc productivity of the enzyme mixture toward powdered chitin was approximately 80% of that observed for colloidal chitin. The ability to directly degrade powdered chitin without acid pretreatment highlights its industrial potential for green and efficient chitin valorization. This enzyme offers a sustainable, pretreatment-free alternative for chitin waste bioconversion into high-value products.

## 1 Introduction

Chitin occurs predominantly in the exoskeletons of crustaceans and insects and is the second most abundant polysaccharides in nature (Schmitz et al., 2019; Elieh-Ali-Komi and Hamblin, 2016). The rapid expansion of seafood processing and insect farming industries generates substantial quantities of chitin-rich waste biomass, including shrimp shells, crab carapaces, insect exuviates, and other processing residues (Qin et al., 2019; Abidin et al., 2017; Ali et al., 2024). This biomass used to be discarded or sent to landfills, leading to environmental concerns such as odor emission, microbial spoilage, and localized pollution. Currently, it is increasingly valorized through controlled degradation to produce high-value derivatives, including chito-oligosaccharides and glucosamine (Elieh-Ali-Komi and Hamblin, 2016; Qin and Zhao, 2019; Karim et al., 2024). Chito-oligosaccharides possess a range of biological activities, including antimicrobial, antioxidant, and anti-inflammatory properties, making them valuable for applications in pharmaceuticals, agriculture, and food industries (Benchamas et al., 2021). Glucosamine is extensively used as a dietary supplement to support joint health and treat osteoarthritis (Yi et al., 2020). These derivatives not only provide a sustainable solution for chitin waste management but also open up new avenues for the utilization of chitin in various high-value applications (Chakravarty and Edwards, 2022; Yi et al., 2020; Benchamas et al., 2021).

The hydrolysis of chitin into these valuable compounds typically involves enzymatic or chemical methods (Schmitz et al., 2019). At present, chemical hydrolysis using hydrochloric acid is predominantly employed in chitin hydrolysis for industrial applications, which allows for the rapid and complete breakdown of chitin into its monomeric components (Abidin et al., 2017; Ali et al., 2024). However, chemical hydrolysis poses significant drawbacks, particularly the generation of hazardous byproducts and water pollution (Ali et al., 2024). It is reported that the production of one ton of glucosamine requires approximately eight tons of 30% (v/v) hydrochloric acid and generates wastewater containing more than 70,000 mg/L of chloride ions, a level far exceeding the recommended freshwater water quality standard for chloride in China ( < 200 mg/L) (Bertuzzi et al., 2018; Hong et al., 2023). These issues highlight the need for robust chitinases to develop greener processes for chitin degradation (Atif et al., 2024).

Microorganisms are well-known for their ability to break down chitin. Various bacteria, fungi, and actinomycetes, such as *Streptomyces griseus, Bacillus subtilis, Serratia marcescens*, and *Acinetobacter calcoaceticus*, *Trichoderma harzianum, Beauveria bassiana, Penicillium oxalicum*, and *Fusarium solani* have been well-studied for their chitinase production (Nakagawa et al., 2020; Malik et al., 2023; Song et al., 2022; Mohiddin et al., 2021; Xie et al., 2021; Das et al., 2024). These chitinolytic enzymes demonstrate a broad range of substrate affinities, optimal pH levels from 4.5 to 9, temperature stability between 30 and 80°C, and differing responses to metal ions (Xie et al., 2021; Cao et al., 2022; Song et al., 2024; Rodriguez et al., 1993; Rodriguez et al., 1995; Pareek et al., 2011). However, these chitinases show limited efficiency against powdered chitin (PCT) as its rigid structure hinders enzyme accessibility (Qin and Zhao, 2019), and swelling of chitin using diluted hydrochloric acid are often required for hydrolysis (Qin and Zhao, 2019; Poria et al., 2021).

We previously isolated and characterized various of fungal species from a mangrove wetland (Han et al., 2023), and found that one of the isolates, identified as *Penicillium oxalicum*, has the ability to breakdown suspended PCT. Enzymes from this fungus has been studied extensively for biotechnological applications such as degradation of plant biomass, shellfish processing, and antimicrobial agents (Rodriguez et al., 1995; Rodriguez et al., 1993; Cao et al., 2022; Song et al., 2024; Pareek et al., 2011; Hu et al., 2024; Liao et al., 2014; Xie et al., 2021). In this study, chitinases from this fungus were isolated and characterized in terms of composition and catalytic activity. The enzyme mixture was evaluated for hydrolysis of powdered chitin under both laboratory and scale-up conditions, and stepwise conversion of chitooligosaccharides to N-acetylglucosamine (GlcNAc) was monitored by TCL and HPLC. This work aimed to assess whether the enzyme mixture could directly hydrolyze powdered chitin without acid pretreatment and to explore its potential for efficient, sustainable chitin valorization.

## 2 Materials and methods

### 2.1 Fungal strain and cultivation media

The mangrove wetland-derived fungal strain, *Penicillium oxalicum* H13, was previously isolated by our lab and deposited in Guangdong Microbial Culture Collection Center (GDMCC No: 64525). The strain was cultured on yeast extract peptone dextrose agar (YPD) supplemented with 5% (w/v) sodium chloride (NaCl), and 1% (v/v) penicillin–streptomycin solution (Corning, China) (Han et al., 2023). Yeast extract peptone broth (YPB) containing 1% (w/v) chitin powder (120 mesh) and 5% (w/v) NaCl was used for all liquid culture, referred as YPB+. Chitin was sterilized using dry heat at a temperature of 170°C for a period of 2.5 h, and then added into autoclaved NaCl contained YPB. The final pH of medium was adjusted to 5.7 to support fungal growth.

### 2.2 Agar plate assay of chitinase

To prepare chitin agar plates, chitin was ultra-micro grinded, passed through a 120-mesh sieve, dry-heat sterilized at 170°C for 1 h using an oven (BXH-65G, Boxun, China), and added into YPD medium as a carbon source in place of dextrose. Congo red was added once the medium cooled to 50°C to visualize zones of chitin degradation. *P. oxalicum* H13 was inoculated at the center of the plate, and its growth was observed regularly. A chitinase producing fungus, *Streptomyces griseus* (kept in local lab) was used as a positive control (Lv et al., 2021).

### 2.3 Cultivation conditions

The fungal strains were cultured on agar plate at 36°C for 7–10 days to attain sufficient conidiation. Conidia were harvested by gentle agitation into a solution containing 0.9% (w/v) NaCl and 0.01% (v/v) Tween 80, filtered through an autoclaved 5 mL tip packed with cotton wool to remove hyphal fragments, and counted using a Neubauer hemocytometer. Liquid cultures were performed in 250 mL conical flasks containing 50 mL of YPB+, inoculated 2 × 10^4^ conidia/mL and incubated at 36°C for 5 days on an orbital shaker with three individual flasks dedicated for each time point. The culture supernatant was collected by centrifugation at 5,000 × *g* for 15 min at 4°C at every 24 h. Fungal biomass was dried and weighed. Data were analyzed using SAS (version 9.1), and mean differences were compared using Duncan’s test at a 0.05 significance level.

### 2.4 Isolation and identification of chitinolytic enzymes

The fungal culture supernatant was ultrafiltered through a Regenerated Cellulose (RS) membrane with Molecular Weight Cut Off (MWCO) filters, where filtrate from 10 kDa MWCO filter and the retentate from 200 kDa MWCO filter were separated to remove large and small molecules. The ultrafiltration retentate was subjected to purification using Sephadex G-100 gel filtration following the method from Du et al. (2021). Chitinase activity containing fractions were pooled together and dried by lyophilization.

The lyophilized powder was resuspended in 50 μL ammonium bicarbonate buffer (100 mM, pH 7.8), reduced using 50 mM dithiothreitol and alkylated using 50 mM iodoacetamide following the method of Snashall et al. (2023). Protein concentration was determined by the Bradford assay and 15 μg of protein from each triplicate sample was subjected to in-solution digestion using trypsin at a protease-to-protein ratio of 1:30 (w/w) at 37°C overnight (Sequencing grade modified, Promega, United States) (Snashall et al., 2023). The resulting peptides were desalted and concentrated using C18 zip-tips (Millipore, China) and subjected to label-free quantitative proteomic analysis using reversed-phase nanoscale liquid chromatography coupled with tandem mass spectrometry (nanoLC−MS/MS) on an LTQ-XL ion-trap mass spectrometer (Thermo, United States) (Grinyer et al., 2004). MS/MS data acquisition and analysis were carried out using Xcalibur software v2.06 (Thermo, United States) (Mirzaei et al., 2014).

The raw result files were converted to mzXML format, and peptide sequences derived from the mass spectra were searched against *P. oxalicum* database using the Global Proteome Machine software with the X! Tandem algorithm (GPM)^1^ (Li et al., 2022). Proteins consistently identified across all three biological replicates and with a minimum total spectral count of five were considered valid identifications (Monavarfeshani et al., 2013). All candidate chitinase proteins were further examined in the dataset and reported if supported by at least one unique peptide with a statistically significant match, ensuring no chitinase was overlooked.

### 2.5 RT-qPCR validation

The gene sequences of the candidate chitinase proteins were retrieved from NCBI (Gene ID: 74441033, 74434174, 74435342, 74441231, 74432796, 74436965, and 74432828). Primers were designed using the Primer-BLAST tool^2^ and synthesized by Sangon Biotech (Shanghai, China). Total RNA (1 μg) was treated with DNase and reverse-transcribed with SuperScript IV (Invitrogen, USA). qPCR reaction was performed on a CFX96 PCR Detection System (Bio-Rad, United States) with SYBR Green Master Mix (Thermo Scientific, United States). Amplification was carried out for 30 cycles in biological triplicate at 94°C for 45 s, 58°C for 45 s, and 72°C for 90 s with three technical replicates. Ct values were averaged across triplicates, and relative abundance was calculated by ΔCt method with actin as reference genes according to the study of Zhao et al. (2016). Mean mRNA relative abundance were compared with normalized iBAQ ratios from LC-MS/MS, and the relationship was evaluated by Pearson correlation using Origin 2024 (OriginLab, United States), verifying coherent regulation between transcript and protein expression.

### 2.6 Chitinolytic activity assay

Chitinolytic activity against powdered chitin (PCT) was measured using a dinitrosalicylic acid (DNS) colorimetric assay (Hussin and Ab Majid, 2020). Briefly, 0.1 mL of enzyme solution was mixed with 0.2 mL of 1% (w/v) chitin powder and 0.3 mL PBS (0.03 mol/L, pH7.2), and then incubated at 45°C for 30 min. Then, 0.4 mL of DNS reagent was added to react with the reducing sugars produced from the hydrolysis. The DNS reagent was prepared by dissolving 10 g of 3,5-dinitrosalicylic acid in 150 mL of 2 M sodium hydroxide, adding 300 g of sodium potassium tartrate, and diluting the solution to a final volume of 1 L with distilled water. Upon heating at 95°C for 10 min, the solution developed a reddish-brown color. Once cooled to room temperature, the undigested, insoluble chitin was removed by centrifugation at 10,000 g for 5 min, and the absorption of the supernatant was measured at 540 nm. The reaction without addition of enzyme was used as a blank control. One unit of chitinase activity (U) was defined as the amount of reducing sugar (μg) released by milliliter enzyme per min under assay condition. Specific activity was calculated as enzyme activity (U/mL) divided by protein concentration (mg/mL), expressed as U/mg (Lv et al., 2019).

### 2.7 Biochemical characterization of the chitinolytic enzymes

The optimal temperature of isolated chitinolytic enzymes against PCT was determined in PBS from 30 to 80°C. The optimal pH was measured at optimal temperature and in 50 mM different buffers containing sodium citrate/citric acid buffer (pH 3.0–6.0), sodium phosphate buffer (pH 7.0–8.0), and Tris/HCl buffer (pH 9.0–10.0), respectively. The effects of ions on the enzyme activity were assayed in the presence of KCl, MgCl_2_, ZnCl_2_, FeCl_3_, CaCl_2_, CuCl_2_ respectively with a content of 0.01–0.5 mM under optimal conditions with no-addition of these chemicals as 100% activity. The thermo-stability profile of the purified chitinase was determined by measuring the enzyme activity after storage at 4–65°C for up to 8 h with 4°C as 100% activity.

The chitin before and after enzymatic hydrolysis was collected by filtration, washed 5 times using ddH_2_O, and subsequently spin-coated onto a silica substrate. The samples were mounted on metallic studs using carbon adhesive tabs, sputter-coated with platinum using a Bal-tec SCD 050 sputter coater, and then analyzed in a Field Emission Scanning Electron Microscope (SEM, JSM-6500F, Japan).

Thin-layer chromatography (TLC) was used to determine the extent of enzyme activity. Reaction mixtures containing 2 mM of chitooligosaccharides (COS, degree of polymerization ranging from 2 to 6, DP 2–6) and 5 μg/mL of purified chitinase in PBS were incubated at 40°C with continuous shaking at 500rpm for 0, 5, 15, 30, 60 min, respectively. The mixtures were then boiled in a water bath for 5 min. After cooling to room temperature, the mixtures were subjected to ready-to-use TLC plates (HPTLC Silica gel 60 F254, Merck, Germany). The plates were then placed in a developing chamber with a solvent system of g n-butyl alcohol/methanol/25% ammonia solution/water at the ratio of 5:4:2:1 (v/v/v/v). Once the solvent front approached the top, the plates were removed and dried using a hair dryer. The dried TLC plate was sprayed evenly with ninhydrin solution (0.2 g ninhydrin in 100 mL absolute alcohol) in an operating fume hood. The TLC plate was heated to about 120°C for approximately 3–5 min, where pink spots corresponding to the migrated oligosaccharides should be visible on a white background under white light.

### 2.8 Conversion of chitin powder in large scale

The potential of *P. oxalicum* H13 chitinase for the enzymatic conversion of dried pre-equilibrated chitin powder to reduced sugar was assessed in 50 L batch reactors in three individual reactors. The reactors containing 0.25 g/L of enzymes and 10 g/L chitin powder in PBS were incubated at 65°C with gentle stirring. Fifty ml of reaction mixture were sampled at designated time points. The mixture was concentrated onto pre-weighed 0.45 μm nylon membranes by vacuum filtration, and washed with 1 L de-ionized water to separate any soluble products, and completely dried at 60°C under vacuum ( ≤ 5 mbar, 24 h), and cooled in a desiccator before re-weighing. The soluble oligosaccharides in the filtrate were combined, dried, resuspended in 50 mL de-ionized water, and analyzed by high-performance liquid chromatography (HPLC) using a Waters 2695 system (Waters, Milford, MA, United States) with a Spherisorb 5 μm particle column (4.6 × 250 mm, Waters). The mobile phase consisted of acetonitrile and Milli-Q water (74:26, v/v) and was delivered isocratically at a flow rate of 0.8 mL/min. Samples were injected at a volume of 10 μL, and oligosaccharides were quantified by UV detection at 195 nm. Yields of reduced sugar were calculated by external calibration against DP1–DP5 and integration of the corresponding HPLC peaks following the method of Lv et al. (2019). This 50 L scale test was also carried out using colloidal chitin, and the differences in hydrolysis yield between powdered and colloidal chitin were evaluated based on dry weight of the remaining insoluble chitin material. All measurements were performed in triplicate and were reported as mean ± standard deviation.

## 3 Results and discussion

### 3.1 Growth and chitinolytic enzyme production of *P. oxalicum* H13

**FIGURE 1:**
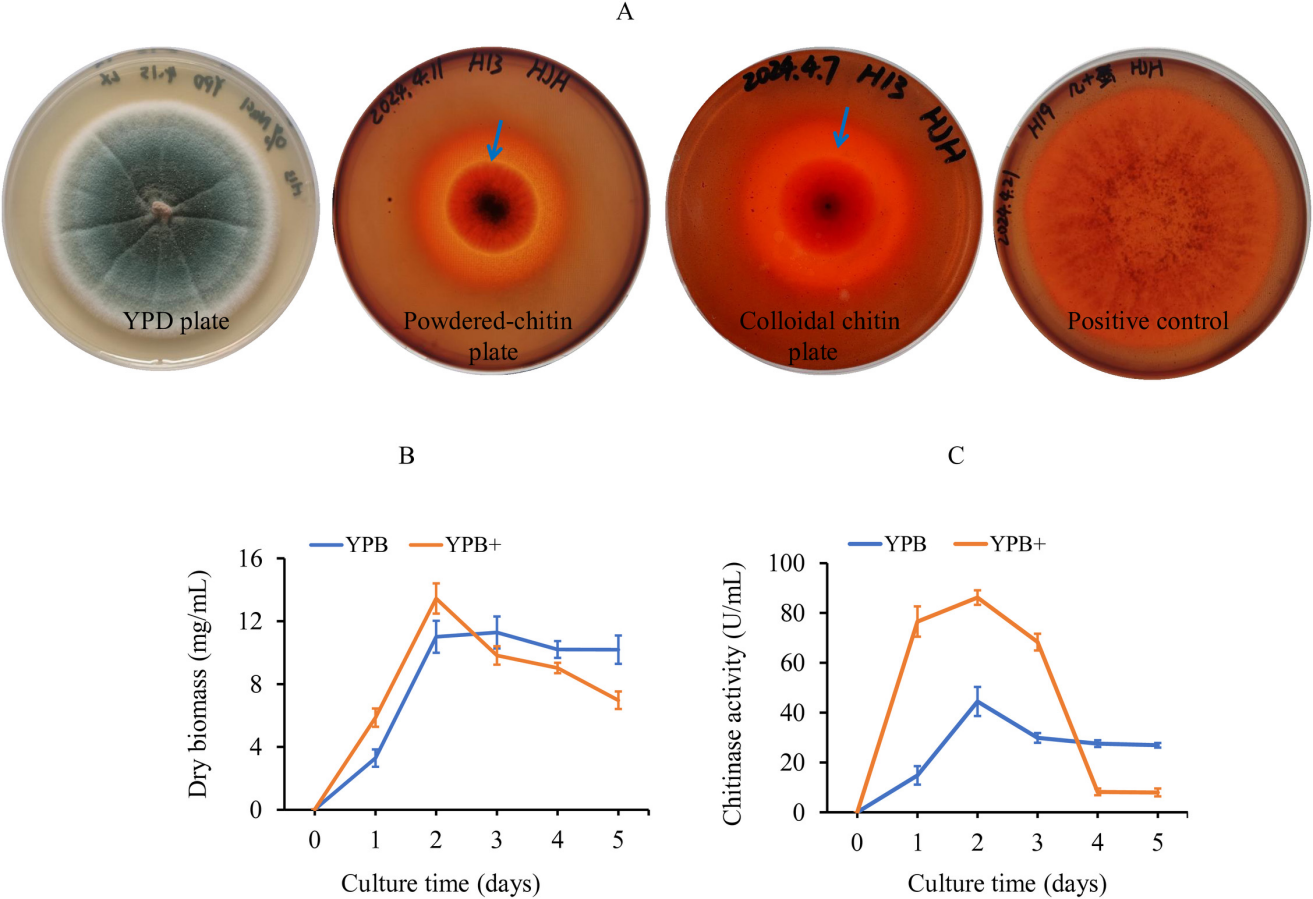
Plate assay and production of chitinases in *P. oxalicum* H13. Plates were incubated at 36^1⁣°^C for 4 days. **(A)** *P. oxalicum* H13 grown on regular YPD medium (left), YPD containing powdered chitin (the second from the left), YPD containing colloidal chitin (the third from the left), and the positive control, *S. griseus*, grown on YPD containing colloidal chitin. Blue arrow indicated the clearing zones resulting from chitinase activity. **(B,C)** Production of biomass and chitinases of *P. oxalicum* H13 grown in regular YPB and powdered chitin containing YPB (YPB+), respectively. Data are presented as mean ± SD of three biological replicates.

A chitin agar is used to assess the ability of *P. oxalicum* H13 to digest chitin, using the anionic azo dye Congo red, a zone of clearance will be observed as the chitin gets digested by the chitinases. As seen from Figure 1A, *P. oxalicum* H13 had a larger clearing zone than the colony size, compared to the positive control, *S. griseus* which showed a clearing zone roughly similar to its colony size after 4 days. This suggests that *P. oxalicum* H13 could secrete chitinase under the tested conditions.

The addition of chitin to the YPB medium (YPB+) altered the growth dynamics of *P. oxalicum* H13 (Figure 1B). Biomass accumulation increased in first 2 days post-inoculation in presence of chitin, mirrored by the increase in chitinase activity (*p* < 0.01) (Figure 1C). Biomass began decreasing after day 2 (Figure 1B), potentially due to the increased chitinase activity killing the fungi by degrading the fungal cell walls. Overall, chitin significantly induced both biomass production and chitinase activity in *P. oxalicum* H13 compared with the YPB (*p* < 0.05).

### 3.2 Identification of chitinolytic enzymes

**FIGURE 2:**
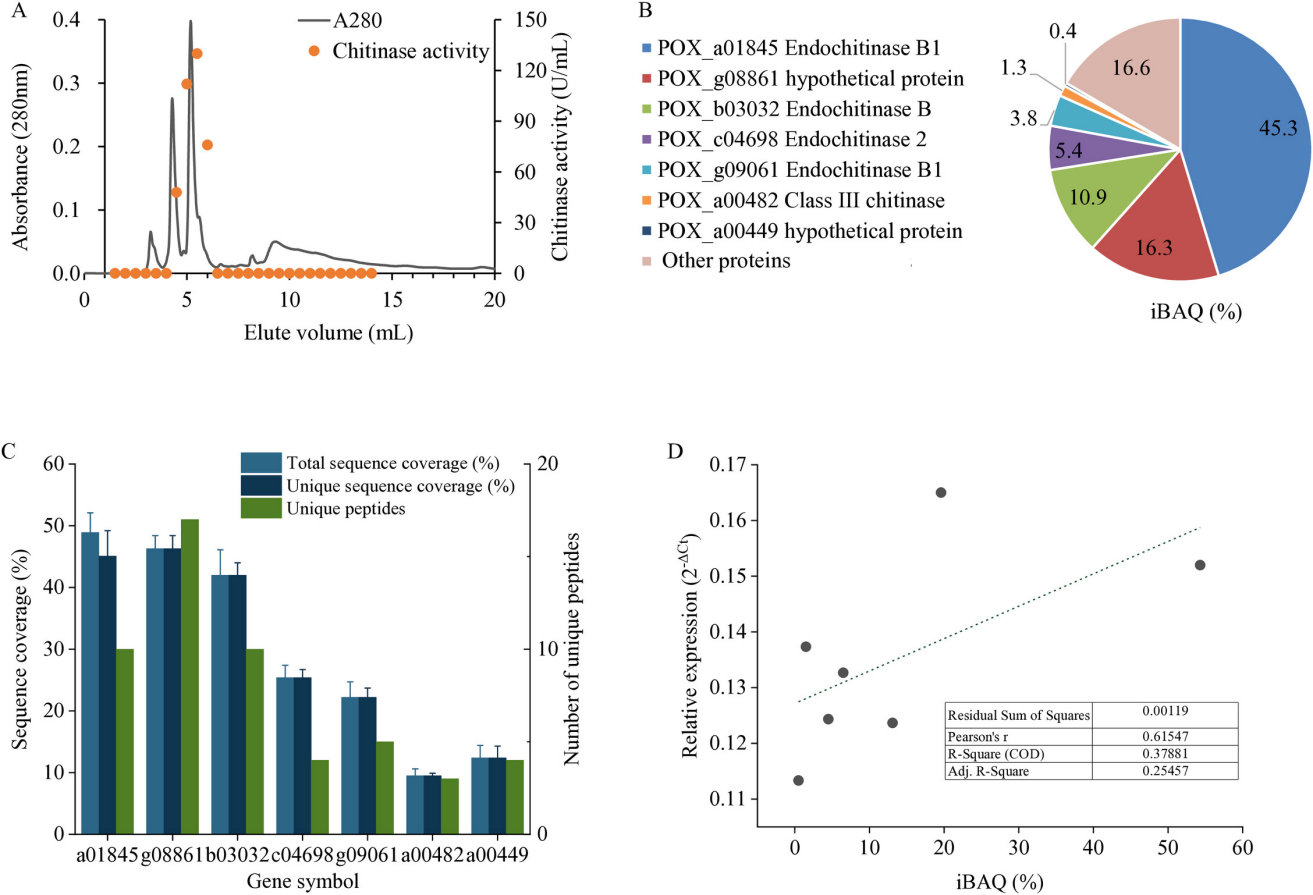
Expression and correlation analysis of secreted chitinases identified from *P. oxalicum* H13. **(A)** Chromatographic separation by gel filtration and chitinase activity assay. **(B)** Gene symbol, description and relative abundance of seven secreted chitinases identified by LC-MS/MS. **(C)** LC-MS/MS identification quality for each chitinase. **(D)** Correlation between transcript and protein abundances. Data are presented as mean ± SD of three biological replicates.

To isolate the chitinolytic enzymes, liquid culture supernatant of the *P. oxalicum* H13 was subjected to gel filtration (Figure 2A), and the two major peaks were identified with chitinase activity; these fractions were used for all the subsequent assays. Purification yield and the purity of final product were investigated (Supplementary Table S1 and Supplementary Figure S1). Proteomics analysis identified seven secreted chitinases (Figure 2B and Supplementary Table S2), accounting for 83.4% of the total abundance quantified using iBAQ, from 3 to 17 unique peptides each (Figure 2C). To further validate the identified chitinases, qRT-PCR was performed targeting the mRNA of the identified proteins. A moderate positive correlation was observed between transcript and protein abundances of the identified chitinases (Pearson’s *r* = 0.61, *R*^2^ = 0.38) (Figure 2D). While the R2 value is not high, the presence of both the transcript and protein ID provides good evidence that these chitinases were indeed expressed by our fungal isolate *P. oxalicum* H13.

### 3.3 Biochemical properties of *P. oxalicum* H13 chitinases

**FIGURE 3:**
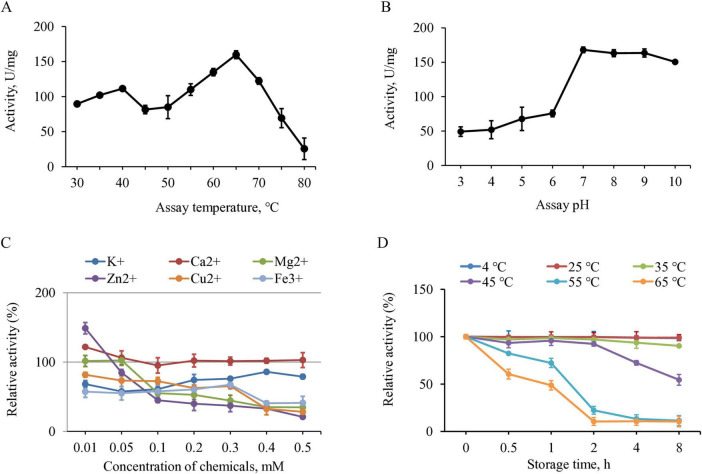
Enzymatic properties of chitinase fraction from *P. oxalicum* H13 using chitin powder as a substrate. **(A)** Temperature-activity profile. **(B)** pH-activity profile at 65°C. **(C)** Effects of metal ions on the enzyme activity. **(D)** Temperature-stability profile. All activity values were expressed as specific activity. Data are presented as mean ± SD of three biological replicates.

At scale, it would not be practical to isolate the individual chitinases from the fungi for breaking down chitin, hence the biochemical properties of the secreted chitinases were assessed as a mixture. A search of the NCBI database revealed that microorganisms produce multiple isoforms of chitinases, each characterized by distinct activity profiles (Li et al., 2022). The majority of these chitinases are active over 30–60°C, with the optimal usually in the 40–50°C range, while thermostable chitinases are optimal at 60–70°C (Lv et al., 2021; Atalla et al., 2020; Thakur et al., 2023; Alves et al., 2018; Lv et al., 2019). For *P. oxalicum* H13, the chitinolytic activity fluctuated with increasing temperature, displaying two maxima at 40°C and 65°C, with the latter showing approximately 1.5-fold higher activity (Figure 3A). The pH profiles of microbial chitinases vary quite broadly across species, sources and enzyme families, spanning from strongly acidic (∼pH 3) to alkaline (pH 10+) (Ren et al., 2022; Alves et al., 2018). The activity of *P. oxalicum* H13 chitinases was higher at neutral pH, and remained stable in alkaline environments (Figure 3B).

The effects of various metal ions on the activity of *P. oxalicum* H13 chitinases were consistent with previous reports on microbial chitinases. Low concentrations of Ca^2+^ and Zn^2+^ ( < 0.01 mM) enhanced chitinolytic activity (Figure 3C), an observation previously reported for *Trichoderma* and *Talaromyces* chitinases (Wang et al., 2023; El-Beltagi et al., 2022). In contrast, Cu^2+^, Fe^3 +^, K^+^, and Mg^2+^ consistently reduced chitinase activity (Figure 3E), consistent with previous reports on fungal chitinases such as AfChiJ, Ta-CHI42, and *P. oxalicum* k10 chitinase (Luong et al., 2021; Xie et al., 2021; He et al., 2022). Overall, these results indicate that *P. oxalicum* H13 chitinases exhibit similar activity profile toward metal ions with fungal chitinases. In addition, *P. oxalicum* H13 chitinases were differentially inhibited by known chitinase inhibitors (Table 1) and its inhibition pattern was similar to *S. griseus* chitinases, with strong sensitivity to argadin and relatively weak inhibition by piperine.

**TABLE 1:** Effects of inhibitors on fungal chitinases.

Inhibitors	Final concentration	Enzyme sources	Residual activity (%)
Allosamidin	10 μmol/L	This study	52.3 ± 6.8
		*Streptomyces griseus*	35.7 ± 3.4
Argadin	10 μmol/L	This study	7.2 ± 1.9
		*Streptomyces griseus*	9.1 ± 1.7
Piperine	100 μmol/L	This study	81.5 ± 5.4
		*Streptomyces griseus*	90.2 ± 2.6

Data = mean ± SD.

The chitinases from *P. oxalicum* H13 demonstrated great thermal stability, retaining almost 100% initial activity after 8 h of storage at 4, 25, and 35°C (Figure 3D). Its activity began to decline at 45°C, with noticeable loss after 2 h. At higher temperatures, approximately 75 and 50% of activity were lost after 1 h at 55°C and 12 h at 65°C, respectively. This temperature profile was similar with that of chitinases from thermophilic fungi such as *Thermomyces lanuginosus* (Suryawanshi and Eswari, 2022), *Rhizopus oryzae* (Chen et al., 2013), and certain strains of *Trichoderma* (Chung et al., 2022) which exhibit optimal temperatures between 55 and 60°C and maintain thermostability up to 50°C (Karthik et al., 2014; Mathew et al., 2021), similar to *P. oxalicum* H13 chitinases. These findings suggest that *P. oxalicum* H13 chitinases could be well-suited for applications demanding activity and stability across a broad range of temperature conditions.

**FIGURE 4:**
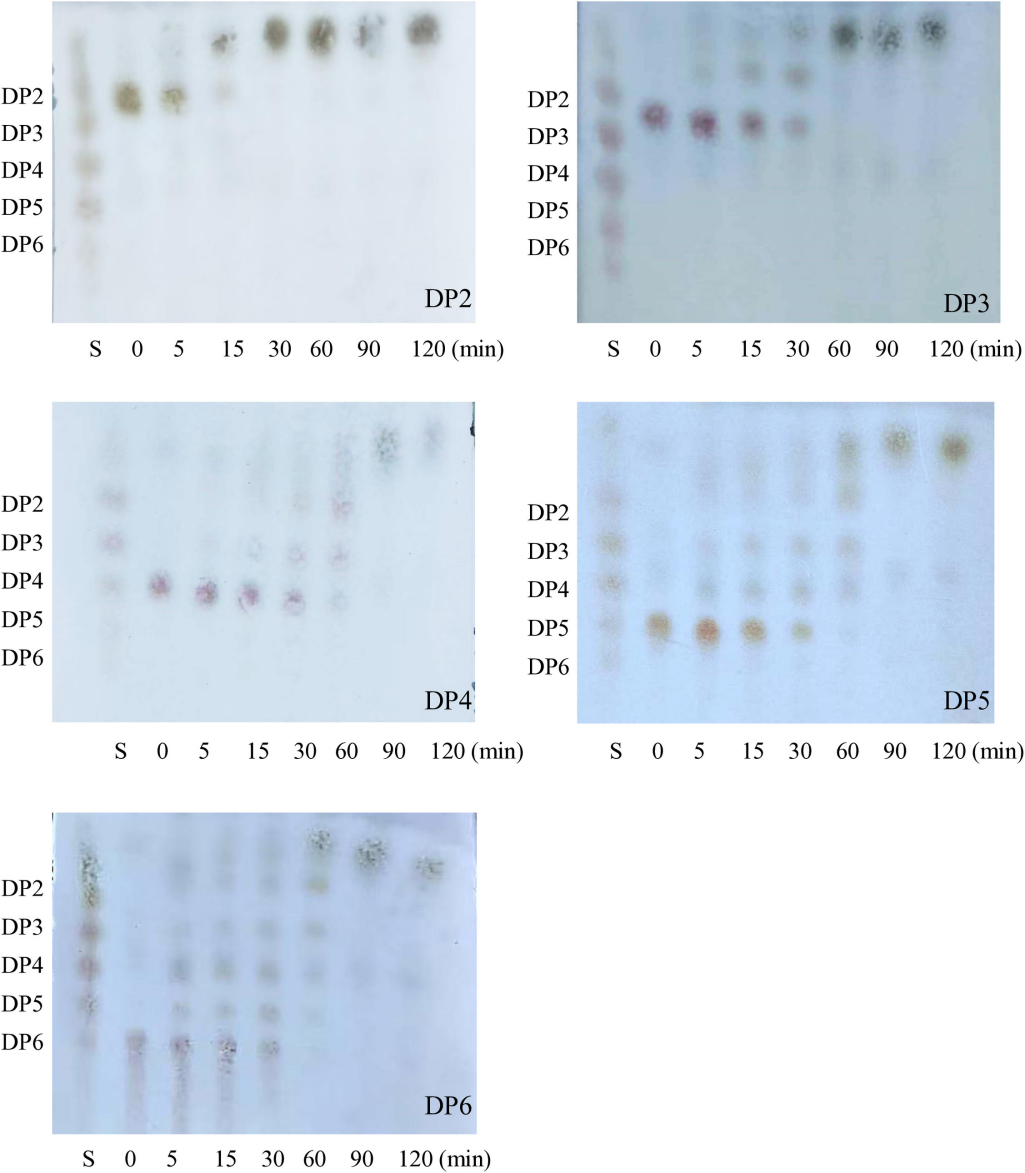
Time courses of COS hydrolysis by *P. oxalicum* H13 chitinase as analyzed by TLC. The reaction mixtures containing 2 mM CHOS of DP 2–6 and 5 μg/mL of purified chitinase in PBS were incubated at 65°C for 0, 5, 15, 30, 60, 90, and 120 min, respectively. Lane: S, the standards of CHOS of DP1–DP6.

Using chitin standards of different lengths (DP2–DP6), the mode of action for *P. oxalicum* H13 chitinases were assessed using thin layer chromatography. Hydrolysis of all substrate lengths tested could be observed within 5 min, indicated by the diminished intensities of higher-degree oligomers (Figure 4). Progressive depletion of DPs occurred from 15 to 90 min, accompanied by transient accumulation and subsequent decline of intermediate oligomers, with the entire substrate pool ultimately converted to N-acetylglucosamine monomers (DP1) by 120 min. Higher-DP oligomers required longer times for complete degradation, about 30 min for DP2, 60 min for DP3, and 90 min for DP4-6. The chitinase exhibited detectable hydrolysis toward both chitobiose (DP2) and higher-DP oligomers and the extent of hydrolysis increased over time, suggesting that the enzyme mixture possessed both endo-type and exo-type catalytic activities (Chung et al., 2022).

### 3.4 Bulk hydrolysis of chitin powder

**FIGURE 5:**
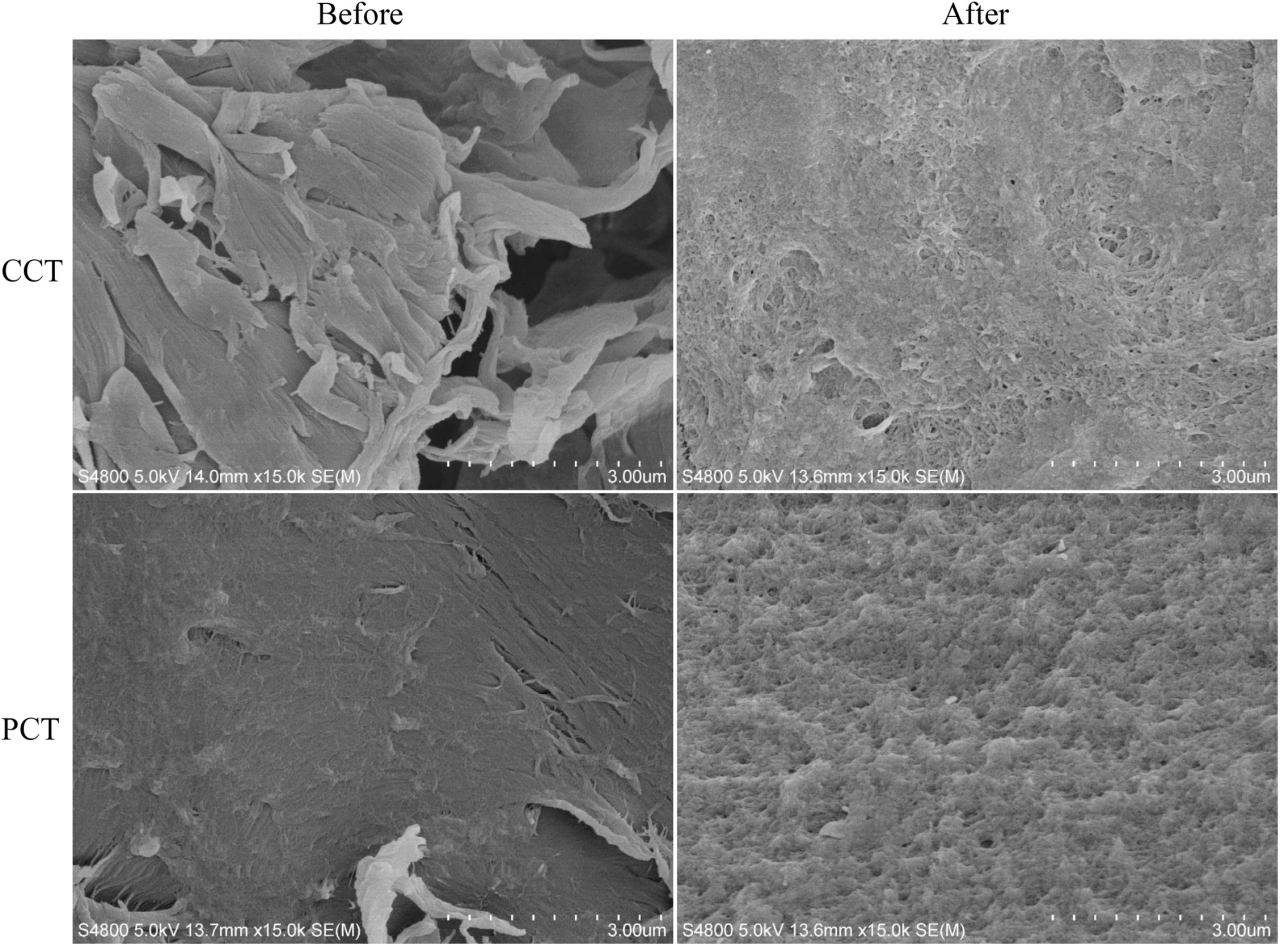
SEM images of PCT and CCT before and after enzymatic hydrolysis by *A. oxalicum* H13 chitinase. Magnification 15,000×.

Pretreatment of chitin using acid, alkaline, mechanical forces, and ionic liquids can decrease the crystallinity of chitin, making it more accessible for enzymatic or chemical hydrolysis (Li et al., 2019; Pohling et al., 2022; Sampath et al., 2022; Poshina et al., 2018). However, these pretreatments may pose environmental risks and reduce equipment longevity. Enzymatically hydrolyzing chitin directly can reduce the release of chemicals and streamline the overall process, providing a simpler and more sustainable option (Li et al., 2019; Pohling et al., 2022). For the *P. oxalicum* H13 chitinases, it was observed to “smoothen” the rough features of CCT, and can also breakdown the smoother surface of the crystalline chitin (PCT) structure when observed using TEM (Figure 5). Studies on various single or combined chitinases have demonstrated GlcNAc yields ranged from 14 to 95% (of theoretical GlcNAc content in the acid-pretreated CCT) over time spans from 6 h to 10 days (Lv et al., 2019; Lv et al., 2021; Nguyen-Thi and Doucet, 2016; Sashiwa et al., 2002; Cardozo et al., 2017; Kuk et al., 2005).

**FIGURE 6:**
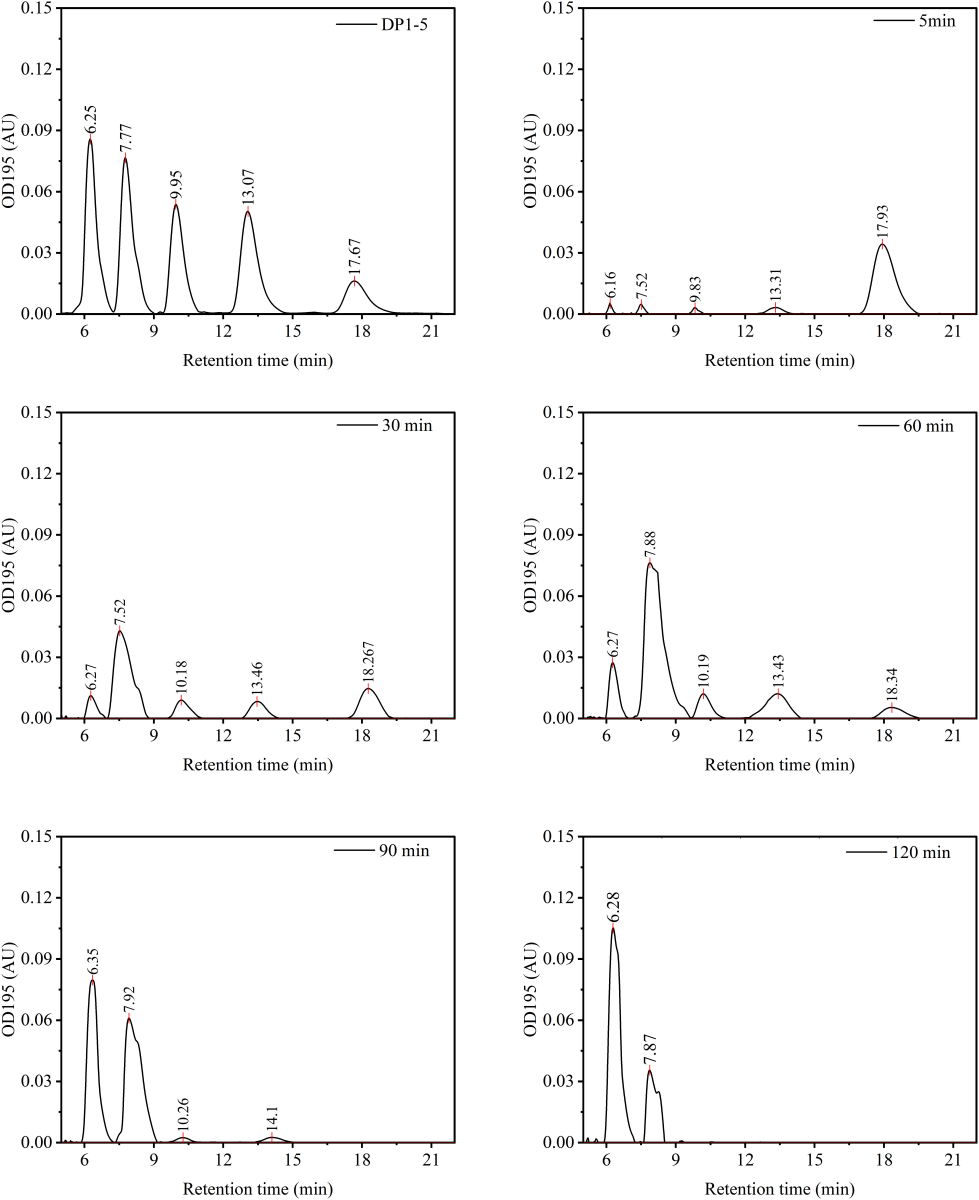
Time courses of chitin powder hydrolysis by *P. oxalicum* H13 chitinase as analyzed by HPLC. The conversion was conducted in 10 L reactors containing 0.25 g/L of the enzyme and 10 g/L chitin powder in PBS at 65°C with gentle shaking. A mixture of DP 1–5 was used as a standard to visualize the hydrolysis products. Labels in the upper-right corner of each panel indicate the standards or the corresponding hydrolysis time.

Activity of *P. oxalicum* H13 chitinase mixture was subsequently examined at scale (50 L) on direct hydrolysis of PCT. Sampling of the reactor supernatant showed effective hydrolysis of chitin into chitooligosaccharides observed by HPLC (Figure 6), and stepwise hydrolysis of chitin similar to the lab-scale test (Figure 5). DP5 chito-oligosaccharides were observed within 5 min and by 30 min, DP2 chitobiose was the major product observed. By 120 min, DP1 was the major product observed, indicating a near-complete polymer degradation, similar to the lab-scale test (Figure 5), further suggesting that the chitinase fraction contained both endo- and exo-type catalytic activities. The yield of reducing sugars eventually reached around 70%, as determined by the dry weight of the remaining insoluble chitin content in the 50 L bioreactor, and peak area quantitation from the HPLC showed that over 60% of the soluble products consisted of DP 1 (Figure 6). When the substrate was changed to CCT, the DP1 yield increased to 77%, indicating that the DP1 productivity of the enzyme mixture toward PCT was around 80% of that toward CCT (Table 2). This result places crude H13 cocktail in the top quartile of wild-type fungal systems since most chitinase preparations worked on CCT or got a low conversion rate under comparable substrate loadings (Table 2).

**TABLE 2:** Representative enzyme driven chitin-to-GlcNAc conversions reported in the last 10 years.

System	Scale	Substrate	Time	Temp.	GlcNAc yield (%)	References
Enterobacter EcChi1	Lab study	CCT	12 h	40°C	37 %	(Mallakuntla et al., 2017)
ScHEX and chitinase C from *S. coelicolor* A3(2)	Lab study	CCT	8 h	50°C	95 %	(Nguyen-Thi and Doucet, 2016)
Recombinant ChiA + BsNagZ	Lab study	CCT	12 h + 20 min	60°C	88 %	(Song et al., 2020)
Chitinase from *Streptomyces speibonae* TKU048	Lab study	PCT	96 h	60°C	73.6%	(Tran et al., 2019)
Recombinant TvChi1 and CmCh i1	Scaled-up	PCT	12 h	53°C	11%	(Yu et al., 2022)
This work	Scaled-up	PCT	2 h	65°C	60 %	–
		CCT	2 h	65°C	77 %	

## 4 Conclusion

This study establishes a scalable biocatalytic process for direct valorization of raw chitin powder without chemical pretreatment. The *P. oxalicum* H13 chitinase mixture achieved efficient GlcNAc production from PCT within 2 h at 65°C on a 50 L scale, delivering approximately 80% of the productivity observed with acid-pretreated substrates. HPLC analysis revealed a clear stepwise hydrolysis pattern, confirming the progressive conversion of chitin into chitooligosaccharides and monomeric GlcNAc. This natural enzyme consortium thus offers a practical and sustainable approach for direct chitin valorization, eliminating the need for harsh acid pretreatment typically required for PCT and offering significant potential for industrial applications in chitin valorization and biorefinery processes.

## Data Availability

The original contributions presented in the study are included in the article/Supplementary material, further inquiries can be directed to the corresponding authors.

## Footnotes

1
http://www.thegpm.org

2
https://www.ncbi.nlm.nih.gov/tools/primer-blast/

## Supplementary material

Supplementary Table S1 Results of purification of chitinase fraction from *P. oxalicum* H13.

Supplementary Table S2 Summary of identification of purified *P. oxalicum* H13 chitinase.

Supplementary Figure S1 SDS-PAGE and zymogram analysis of the *P. oxalicum* H13 chitinase mixture. Left panel: SDS-PAGE, lane 1: culture supernatant, lane 2: retentate from 10 kDa MWCO filter, lane 3: filtrate from 200 kDa MWCO filter, lane 4: enzymes after gel filtration. Right panel: zymogram, lane 1: culture supernatant, lane 2: enzymes after gel filtration.
