# Supplementary material for: Efficacy of chitinases from mangrove wetland derived Penicillium oxalicum on powdered chitin
Source: Front Microbiol. 2026 Mar 18;17:1773725. doi: 10.3389/fmicb.2026.1773725 (PMC13038932; doi:10.3389/fmicb.2026.1773725)
Supplement: Supplementary Table S2 — Summary of identification of purified P. oxalicum H13 chitinase. [file Table_2.docx]

Table S2. Results of purification of chitinase fraction from *P. oxalicum* H13

| Purification stage | Total enzyme activity (U) | Total protein (mg) | Enzymatic specific activity（U/mg) | Purification ratio | Recovery percent（%） |
| --- | --- | --- | --- | --- | --- |
| crude enzyme | 3461.56±109.87 | 887.91±37.58 | 3.90±0.24 | 1.00 | 100 |
| Ultrafiltration | 1890.87±57.54 | 201.04±19.83 | 9.41±0.61 | 2.42±0.35 | 54.62±4.91 |
| Gel filtration | 417.41±20.12 | 7.85±0.64 | 53.17±3.58 | 13.63±1.02 | 12.06±0.87 |
