# Supplementary figures and images for: Efficacy of chitinases from mangrove wetland derived Penicillium oxalicum on powdered chitin
Source: Front Microbiol. 2026 Mar 18;17:1773725. doi: 10.3389/fmicb.2026.1773725 (PMC13038932; doi:10.3389/fmicb.2026.1773725)

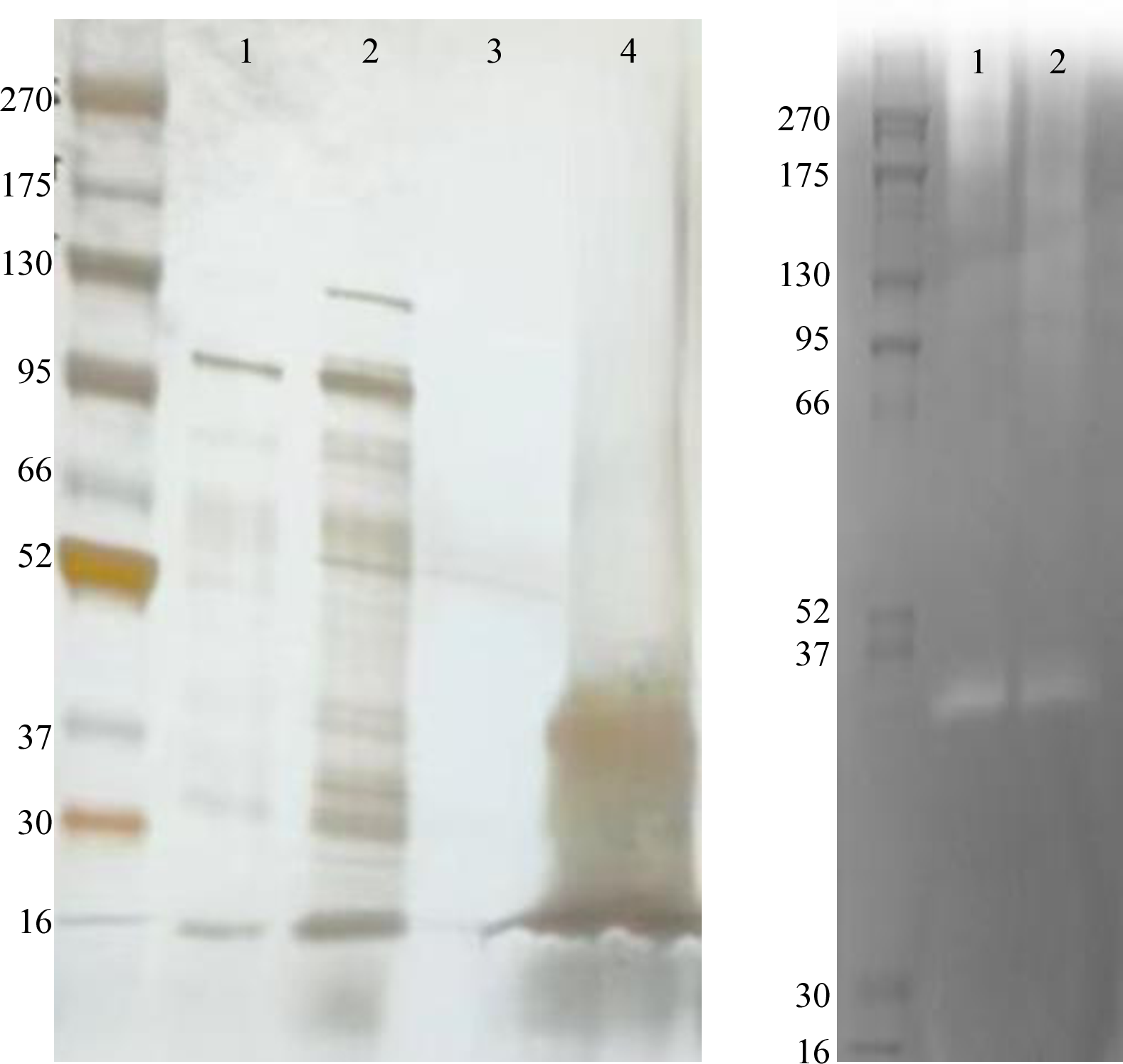

Supplement: Supplementary Figure S1 — SDS-PAGE and zymogram analysis of the P. oxalicum H13 chitinase mixture. Left panel: SDS-PAGE, lane 1: culture supernatant, lane 2: retentate from 10 kDa MWCO filter, lane 3: filtrate from 200 kDa MWCO filter, lane 4: enzymes after gel filtration. Right panel: zymogram, lane 1: culture supernatant, lane 2: enzymes after gel filtration. [file Image_1.tif]
